# Are whooping cranes destined for extinction? Climate change imperils recruitment and population growth

**DOI:** 10.1002/ece3.2892

**Published:** 2017-03-21

**Authors:** Matthew J. Butler, Kristine L. Metzger, Grant M. Harris

**Affiliations:** ^1^ Division of Biological ServicesU.S. Fish and Wildlife ServiceAlbuquerqueNMUSA

**Keywords:** atmospheric CO_2_, boreal pond, decadal cycle, groundwater, LASSO, precipitation, reproduction, solar activity, sunspots

## Abstract

Identifying climatic drivers of an animal population's vital rates and locating where they operate steers conservation efforts to optimize species recovery. The population growth of endangered whooping cranes (*Grus americana*) hinges on juvenile recruitment. Therefore, we identify climatic drivers (solar activity [sunspots] and weather) of whooping crane recruitment throughout the species’ life cycle (breeding, migration, wintering). Our method uses a repeated cross‐validated absolute shrinkage and selection operator approach to identify drivers of recruitment. We model effects of climate change on those drivers to predict whooping crane population growth given alternative scenarios of climate change and solar activity. Years with fewer sunspots indicated greater recruitment. Increased precipitation during autumn migration signified less recruitment. On the breeding grounds, fewer days below freezing during winter and more precipitation during breeding suggested less recruitment. We predicted whooping crane recruitment and population growth may fall below long‐term averages during all solar cycles when atmospheric CO_2_ concentration increases, as expected, to 500 ppm by 2050. Species recovery during a typical solar cycle with 500 ppm may require eight times longer than conditions without climate change and the chance of population decline increases to 31%. Although this whooping crane population is growing and may appear secure, long‐term threats imposed by climate change and increased solar activity may jeopardize its persistence. Weather on the breeding grounds likely affects recruitment through hydrological processes and predation risk, whereas precipitation during autumn migration may influence juvenile mortality. Mitigating threats or abating climate change should occur within ≈30 years or this wild population of whooping cranes may begin declining.

## 1 Introduction

Identifying drivers of animal population demographics (i.e., associations between environmental conditions and vital rates) and locating where they operate is necessary for steering efforts to optimize wildlife species management and conservation (e.g., Caughley, 1994; Mills, 2007). Doing so establishes a foundation to evaluate how climate change may impact an animal's population growth and vulnerability (Brook et al., 2009). This information is especially critical for recovering species endangered with extinction (Akçakaya, Butchart, Watson, & Pearson, 2014). Herein, we focus on the population growth of endangered whooping cranes (*Grus americana*), which hinges on recruitment (Butler, Metzger, & Harris, 2014).

Our aims were to model associations between whooping crane recruitment and environmental conditions, examine how climate change affects those conditions, and translate those effects to population growth. Our work reveals the climatic threats on the species and where they operate, it predicts the prospects for species recovery, and helps develop hypotheses about how climate drivers act on whooping crane demographics and where management intervention would be most effective. We demonstrate that although the wild whooping crane population is growing and therefore may appear secure (Butler, Harris, & Strobel, 2013), long‐term threats imposed by climate change jeopardize this population's persistence (Carroll, Townshend, DiMiceli, Loboda, & Sohlberg, 2011; Chavez‐Ramirez & Wehtje, 2012; Smith, Sheng, MacDonald, & Hinzman, 2005).

In North America, whooping cranes breed in the boreal ecosystem of Canada (Canadian Wildlife Service [CWS] & U.S. Fish and Wildlife Service [USFWS], 2007; Kuyt, 1995). Here, one of the most studied biological phenomena is the approximate decadal cycles of snowshoe hare (*Lepus americanus*) and lynx (*Lynx canadensis*; Krebs, Boonstra, Boutin, & Sinclair, 2001). This decadal cycle is well documented in other species (Klvana, Berteaux, & Cazelles, 2004; Marques, Short, & Creed, 2014; Selås, Hogstad, Kobro, & Rafoss, 2004; Sinclair & Gosline, 1997), occurs in whooping crane demographics (Butler et al., 2013), and appears in ground‐ and surface water levels of the whooping crane breeding grounds (Gibson, Prowse, & Peters, 2006; McNaughton, 1991; Timoney, Zoltai, & Goldsborough, 1997). Recent work confirms that the lynx cycle is inversely correlated with whooping crane fledging rate (Wilson, Gil‐Weir, Clark, Robertson, & Bidwell, 2016), while fledgling rate influences recruitment and ultimately whooping crane recovery. Correlation between solar irradiance (Krivova, Vieira, & Solanki, 2010), as measured by the number of sunspots, to mammal abundance is also apparent (Sinclair & Gosline, 1997). While the proximal regulator of population cycles in boreal species remains unclear, the relationship likely involves complex interactions among food supply, predator abundance, weather patterns and hydrology, which may be influenced by the solar cycle (Krebs & Berteaux, 2006; Krebs et al., 2001; Rind, Lean, Lerner, Lonergan, & Leboissetier, 2008; Sinclair & Gosline, 1997).

Given that the approximate decadal population cycle observed in many boreal species appears mediated through climate processes (Scott & Craine, 1993; Yan, Stenseth, Krebs, & Zhang, 2013), we examined whether and how global climate change may influence whooping crane demographics. Climatic changes may affect whooping crane recruitment through direct (i.e., increases in predator densities) or complex indirect interactions. For example, whooping cranes build nests in shallow lakes and ponds of the taiga to deter predation (Kuyt, 1995; Kuyt, Barry, & Johns, 1992; Timoney et al., 1997). Climate change is reducing the amount of surface water and likely the depth and seasonal longevity of these ponds (Carroll et al., 2011; Smith et al., 2005). Fewer, shallower, and more ephemeral ponds failing to persist through the entire nesting period may increase nest predation, thereby reducing recruitment of juveniles (Timoney et al., 1997).

Other factors outside of the breeding grounds may also affect whooping crane recruitment as it represents a combination of several demographic parameters (e.g., breeding propensity, clutch size, hatch and fledge rates, and juvenile survival during migration). Reproduction by adults can be supported using stored resources (known as capital breeding) or via the uptake of resources concomitant to a reproductive attempt (known as income breeding; Stephens, Boyd, McNamara, & Houston, 2009). If a capital breeding strategy is employed by whooping cranes, weather conditions prior to their departure to the breeding grounds may affect breeding propensity and nest success. Alternatively, juveniles may be influenced by conditions encountered during southern migration (e.g., inclement weather, food quality, and quantity) that could reduce their survival and subsequently affect their recruitment into the winter flock.

We examined environmental drivers of whooping crane recruitment by modeling it as a function of solar, weather, and hydrological factors spanning all portions of its life cycle (breeding, wintering, and migration). On the breeding grounds, these models examined associations of recruitment with the solar cycle and winter temperature and precipitation. For the wintering grounds, models included drought conditions and freshwater balance of estuaries, as these factors may limit food production (Butler et al., 2014). During the spring and fall migrations, models evaluated temperature, precipitation and drought, as each affects food availability and production, and inclement weather may increase juvenile mortality during southern migration (Kuyt, 1992).

We predicted the potential impact of climate change on whooping crane recruitment and population growth for future scenarios, by linking the primary drivers of recruitment with atmospheric CO_2_ concentrations (elevated concentrations are indicative of climate warming) and the solar cycle. Scenarios of atmospheric CO_2_ concentration consisted of the 2015 concentration (≈400 ppm) and the predicted 2050 concentration (≈500 ppm; Intergovernmental Panel on Climate Change [IPCC], 2014). The solar cycle scenarios were the modern grand maximum (1954–1964), the Dalton minimum (1811–1821), and a typical cycle. This procedure reveals how climate change may affect the whooping crane population's dynamics through 2050, provides expectations for species recovery, and indicates where management intervention would likely be most effective.

## 2 Material and Methods

### 2.1 Breeding grounds, wintering grounds, and migratory route

**Figure 1:**
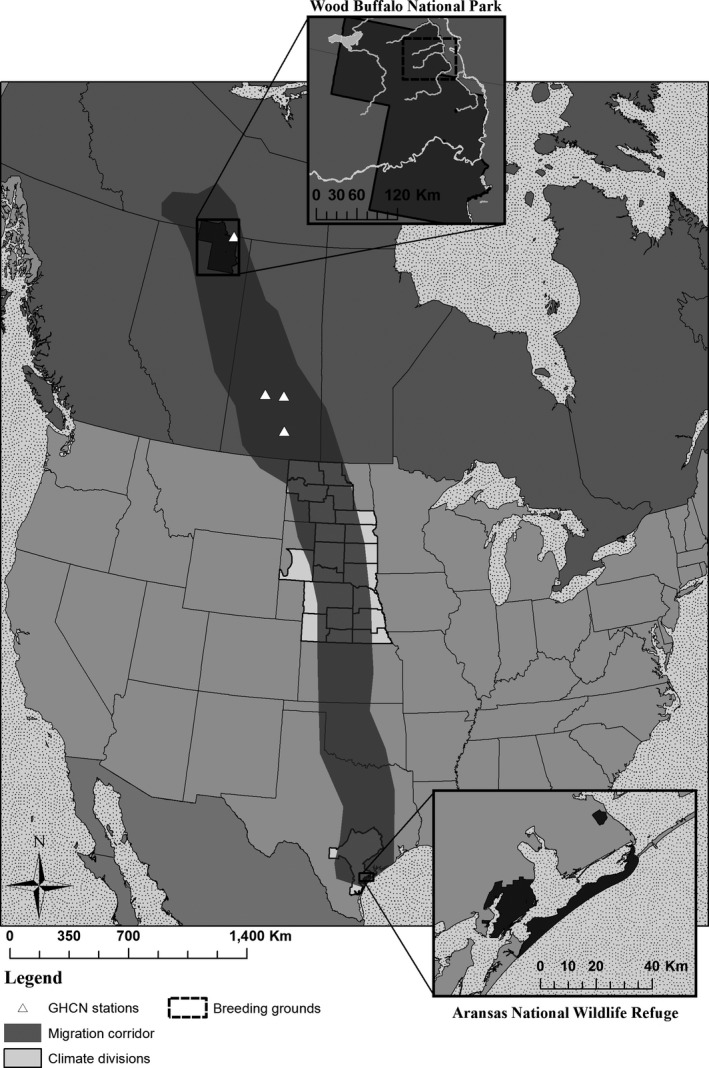
Migratory route of whooping cranes in North America. The breeding grounds are on and around Wood Buffalo National Park, Canada, and the wintering grounds are on and around Aransas National Wildlife Refuge, Texas, USA

Only one wild, migratory population of whooping cranes exists. It breeds on and around Wood Buffalo National Park, Alberta, and Northwest Territories, Canada, and overwinters along the Gulf of Mexico centered on Aransas National Wildlife Refuge (NWR), Texas, USA (≈4,000 km migration, one way; CWS & USFWS, 2007; Figure 1). This population is growing, yet small (≈340 birds), range restricted, and therefore considered endangered with extinction (Butler et al., 2013, 2014; CWS & USFWS, 2007).

Whooping cranes arrive on their wintering grounds surrounding Aransas NWR beginning in October and depart by late April. Their migratory route leads north through central Texas, Oklahoma, Kansas, and Nebraska into western South Dakota and North Dakota to the USA–Canada border (Kuyt, 1992). Their migratory route continues north‐northwest across Saskatchewan to Alberta and then north to Wood Buffalo National Park (Figure 1; Kuyt, 1992).

Whooping cranes begin arriving on their breeding grounds by late April, and their nests are initiated by mid‐May (Kuyt, 1995). Whooping cranes build their nests in shallow boreal ponds or lakes by constructing a mound of mud and vegetation in open water or placing it among clumps of brush on natural islets (Kuyt, 1995; Kuyt et al., 1992; Timoney et al., 1997). Whooping cranes, along with the young of the year, depart the breeding grounds starting in mid‐September and follow the same migratory route south to the Texas coast (Figure 1; Kuyt, 1992). During the autumn migration, central Saskatchewan appears to be a staging area in which whooping cranes congregate and forage on waste grain during late September to mid‐October (Johns, Woodsworth, & Driver, 1997). Once whooping cranes depart the staging grounds in central Saskatchewan, southern migration across the USA can be as short as 1 week with delays occurring due to low‐pressure weather systems (Kuyt, 1992).

### 2.2 Aerial surveys

Annual, repeated aerial surveys of whooping cranes have been conducted by USFWS personnel on the wintering grounds between October and May since 1950 (Butler, Strobel, & Eichhorn, 2016; Strobel & Butler, 2014). The survey has been primarily conducted from a fixed‐wing aircraft with transects spaced approximately 250–800 m apart (Butler et al., 2016). The number of surveys conducted each winter between December 1 and March 31 has ranged from 4 to 21 (Butler et al., 2014). This survey produced counts of total winter abundance and number of juvenile birds recruited plus estimates of annual mortality (Butler et al., 2013, 2014).

### 2.3 Possible predictors of recruitment

As indicators of weather conditions on the wintering grounds, we obtained monthly values of the Palmer drought indices for Texas Climatological Division 7 (Figure 1; Palmer, 1965; National Climatic Data Center [NCDC] 2007). For each of the Palmer indices, we averaged the months of November–March of each year. We obtained monthly freshwater balance (i.e., balance = inflow − evaporation + precipitation) for the Guadalupe and Mission‐Aransas estuaries (Guthrie & Lu, 2010; Schoenbaechler, Guthrie, & Lu, 2011). For both estuaries, we summed the months of November–March of each year and scaled estimates to 1 million acre‐feet. The Aransas‐Wood Buffalo whooping crane population overwinters along both estuaries, so we also combined freshwater balance for them. As environmental conditions on the wintering grounds may affect whooping crane body condition and therefore reproduction once birds return to the breeding grounds (i.e., capital breeding; Stephens et al., 2009), we lagged the wintering ground variables by 1 year (e.g., drought indices from November 1950–March 1951 were paired with recruitment during winter 1951–1952).

We divided the migratory route into two primary areas: northern U.S. Great Plains and southern Saskatchewan prairies. We obtained monthly values for the Palmer drought indices for Nebraska Climatological Divisions 2–9, South Dakota Climatological Divisions 2–3 and 6–9, and North Dakota Climatological Divisions 1–2, 4–5, and 8–9 (Figure 1; Palmer, 1965; NCDC 2007), as indicators of climatic conditions on the northern U.S. Great Plains. For each of the Palmer indices, we averaged data from the Climatological Divisions across months for the spring migration period (i.e., April) and autumn migration period (i.e., October–November).

We used the proportion of days in which the maximum temperature remained below freezing (i.e., ≤0°C), the proportion of days in which minimum temperature was below freezing, mean temperature, and the total precipitation obtained from three Global Historical Climatology Network (GHCN) stations (i.e., Regina University, CA004016640; Saskatoon Airport, CA004057120; and Muenster, CA004015440) located across the southern Saskatchewan prairies (Figure 1). All three of these stations had a complete record from 1950–2010 and covered the southern and northern portions of the migratory route through the southern Saskatchewan prairies. Data were pooled across the three stations and months for the spring (i.e., April) and autumn migration periods (i.e., September–October).

Few long‐term GHCN stations exist in the vicinity of Wood Buffalo National Park and the whooping crane's breeding grounds. We obtained weather data (same measures used for southern Saskatchewan prairies) from the GHCN station at Fort Smith, Northwest Territories, Canada (CA002202200), the station closest to the breeding grounds (Figure 1). For the breeding grounds, we also considered two periods as potentially important to recruitment: winter (November–March) and breeding (April–September). We considered the winter prior to arrival because surface water conditions in breeding ponds may be affected by winter weather (Yoshikawa & Hinzman, 2003).

The solar cycle has been implicated as a potential factor in the cyclic population dynamics of lynx, snowshoe hare, and other organisms (Klvana et al., 2004; Krebs et al., 2001; Marques, Short, et al., 2014; Selås et al., 2004). Sunspots are a long‐term indicator of total solar irradiance (Krivova et al., 2010), so we obtained the annual sunspot number from the Royal Observatory of Belgium (SILSO World Data Center 2015). We used year as a predictor to investigate trends in recruitment.

### 2.4 Addressing bias in recruitment estimates

We suspected recruitment estimates may have been biased high during years with fewer surveys (similar biases occurred with winter mortality estimates; Butler et al., 2014). We examined recruitment estimates for this potential bias by regressing recruitment and the number of surveys conducted during a winter between December 1 and March 31 (logistic regression). If unbiased, recruitment estimates should not be associated with the number of surveys. However, if an inverse relationship between recruitment and the number surveys exists, it indicates some adults are missed when the number of surveys is small. For example, detection of a whooping crane group was estimated as 55.8% (Strobel & Butler, 2014) which means the cumulative detection probably does not exceed 99% until six surveys are completed. Therefore, estimates of recruitment are likely biased high during years in which the number of surveys conducted was less than six. We removed those potentially biased years and reexamined the association between recruitment and the number of surveys conducted.

### 2.5 Modeling recruitment and survival with LASSO

We modeled recruitment (i.e., proportion of juvenile whooping cranes in the total population count for each winter) as a function of the predictor variables described above (Table 1), many of which were collinear, using logistic regression. To select the model, we used the least absolute shrinkage and selection operator (LASSO), a statistical regularization technique (i.e., procedure for balancing model fit and complexity) appropriate for model selection when a relatively large set of possibly collinear predictor variables are used (Gerber, Kendall, Hooten, Dubovsky, & Drewin, 2015; Tibshirani, 1996). Unlike other statistical regularization techniques (i.e., ridge regression), LASSO regression allows some effects to be exactly zero which results in a more easily interpretable model, because the number of predictors is reduced (Reineking & Schröder, 2006; Tibshirani, 1996). In LASSO regression, the balance between model fit and complexity is achieved through the parameter lambda.

**Table 1:** Variables used to model recruitment of juvenile whooping cranes into the winter population

Variable^a^	Description
Global	
SSN	Annual sunspot number
TIME	Sequence of 1 to 61 indicating an annual time step
SURVEYS	Number of aerial surveys conducted between 1 December and 31 March
Wintering Grounds	
PDSI.ANWR.1103	Mean Palmer drought severity index for November–March on Texas Coast
PHDI.ANWR.1103	Mean Palmer hydrological drought index for November–March on Texas Coast
PMDI.ANWR.1103	Mean modified Palmer drought severity index for November–March on Texas Coast
ZNDX.ANWR.1103	Mean Palmer Z index for November–March on Texas Coast
GEFB.ANWR.1103	Total Guadalupe Estuary freshwater balance for November–March
MAEFB.ANWR.1103	Total Mission‐Aransas Estuary freshwater balance for November–March
CoEFB.ANWR.1103	Total combined estuary freshwater balance for November–March
Spring migration	
PDSI.NEDA.04	Mean Palmer drought severity index for April in northern U.S. Great Plains
PHDI.NEDA.04	Mean Palmer hydrological drought index for April in northern U.S. Great Plains
PMDI.NEDA.04	Mean modified Palmer drought severity index for April in northern U.S. Great Plains
ZNDX.NEDA.04	Mean Palmer Z index for April in northern U.S. Great Plains
TPCP.SASK.04	Total precipitation (cm) in southern Saskatchewan prairie during April
DT32.SASK.04	Proportion of days during April in southern Saskatchewan prairie with minimum temperature ≤0°C
DX32.SASK.04	Proportion of days during April in southern Saskatchewan prairie with maximum temperature ≤0°C
MNTM.SASK.04	Mean temperature (F°) during April in southern Saskatchewan prairie
Breeding grounds	
TPCP.WBNP.1103	Total precipitation (cm) during November–March in Wood Buffalo National Park
DT32.WBNP.1103	Proportion of days during November–March in Wood Buffalo National Park with minimum temperature ≤0°C
DX32.WBNP.1103	Proportion of days during November–March in Wood Buffalo National Park with maximum temperature ≤0°C
MNTM.WBNP.1103	Mean temperature (F°) during November–March in Wood Buffalo National Park
TPCP.WBNP.0409	Total precipitation (cm) during April–September in Wood Buffalo National Park
DT32.WBNP.0409	Proportion of days during April–September in Wood Buffalo National Park with minimum temperature ≤0°C
DX32.WBNP.0409	Proportion of days during April–September in Wood Buffalo National Park with maximum temperature ≤0°C
MNTM.WBNP.0409	Mean temperature (F°) during April–September in Wood Buffalo National Park
Autumn migration	
PDSI.NEDA.1011	Mean Palmer drought severity index for October–November in northern U.S. Great Plains
PHDI.NEDA.1011	Mean Palmer hydrological drought index for October–November in northern U.S. Great Plains
PMDI.NEDA.1011	Mean modified Palmer drought severity index for October–November in northern U.S. Great Plains
ZNDX.NEDA.1011	Mean Palmer Z index for October–November in northern U.S. Great Plains
TPCP.SASK.0910	Total precipitation (cm) in southern Saskatchewan prairie during September–October
DT32.SASK.0910	Proportion of days during September–October in southern Saskatchewan prairie with minimum temperature ≤0°C
DX32.SASK.0910	Proportion of days during September–October in southern Saskatchewan prairie with maximum temperature ≤0°C
MNTM.SASK.0910	Mean temperature (F°) in southern Saskatchewan prairie during September–October

a The first four letters of each variable's name represent the weather index and follow the standard acronym used if available. The second four letters are acronyms for location (e.g., ANWR = Aransas National Wildlife Refuge, WBNP = Wood Buffalo National Park, SASK = Saskatchewan, NEDA = Nebraska and the Dakotas). The four numbers indicate the range of months the variable includes (e.g., 1103 represents November–March).

We used the glmnet package (Friedman, Hastie, & Tibshirani, 2010; Hastie & Qian, 2015) in program R (R Core Team 2014) to conduct the analysis. We used the cv.glmnet function to preform tenfold cross‐validation to select the optimal lambda (i.e., most regularized model whereby the mean cross‐validated error is within one standard error of the minimum; Hastie & Qian, 2015). As the folds used in cross‐validation are selected randomly, we repeated the cross‐validation procedure 1,000 times to characterize the uncertainty in lambda and the model coefficients. We also calculated the proportion of repeats in which a predictor variable was selected.

We conducted the repeated cross‐validated LASSO procedure described above for survival as well. We estimated annual whooping crane survival (${\hat{S}}_{t}$) as $1-\left({\hat{N}}_{t}-{\hat{A}}_{t+1}\right)/{\hat{N}}_{t}$, where ${\hat{N}}_{t}$ was the total abundance during winter *t* and ${\hat{A}}_{t+1}$ was the number of after‐hatch‐year birds in the population during winter *t* + 1 (Butler et al., 2014).

### 2.6 Modeling weather variables

The repeated cross‐validated LASSO procedure identified the most important weather variables for predicting recruitment. We modeled those weather variables as functions of sunspot number and the natural log of mean annual atmospheric CO_2_ concentration (μmol of CO_2_/mol [ppm]; Keeling et al., 2001). Simple linear or logistic regression was used depending on the distributional characteristics of each variable. We used data from 1958–2014 for these analyses as measurement of the concentration of atmospheric CO_2_ began in March 1958 (Keeling et al., 2001). By assuming a log‐linear relationship with CO_2_ concentration, we obtain conservative estimates describing the potential magnitude of change.

### 2.7 Relationships with nesting pond depth

Kuyt et al. (1992) measured the water depth of whooping crane nesting ponds during late May 1976–1991 and reported the annual mean. As surface water conditions in boreal ponds are affected by winter temperature and snow depth (Yoshikawa & Hinzman, 2003), we examined these relationships on the whooping crane's breeding grounds. We used simple linear regression to relate mean pond depth to sunspot number and the proportion of freezing days and precipitation on the breeding grounds during November–March. We also used logistic regression to examine the relationship between recruitment and pond water depth. This analysis helped explain associations between some of the weather variables and recruitment.

### 2.8 Population growth, recruitment, survival, and environmental change

To predict the potential impact of climate change on whooping crane recruitment, we assessed the potential changes in climatic condition for various scenarios of atmospheric CO_2_ concentration and sunspot number. First, we developed three solar cycle scenarios: one based on the cycle that contained the modern grand maximum (1954–1964; min = 6.6, max = 269.3), one based on the Dalton minimum (1811–1821; min = 2.3, max = 76.3), and one based on a typical cycle. To characterize the typical solar cycle, we fit the sunspot number (1712–2007) as a polynomial of the number of years since a cycle began. We examined two atmospheric CO_2_ concentration scenarios: the 2015 concentration of ≈400 ppm and the predicted 2050 concentration of ≈500 ppm (IPCC, 2014). We converted predicted recruitment from a proportion to a ratio by proportion/(1– proportion). We estimated predicted population growth ($\hat{\overset{¯}{\lambda }}$) for each scenario as ($\hat{\overset{¯}{S}}$) $\left(1+\hat{\overset{¯}{R}}\right)$ where $\hat{\overset{¯}{S}}$ is the geometric mean of annual survival and $\hat{\overset{¯}{R}}$ is the predicted ratio of hatch‐year to after‐hatch‐year birds for each scenario (Butler et al., 2014).

To characterize the uncertainty in predicted recruitment and population growth rate for the climate change scenarios, we implemented a nonparametric bootstrap procedure. Specifically, for each iteration of the repeated cross‐validated LASSO procedure, we bootstrapped the models for each of the important weather variables and predicted the weather condition for each scenario. We bootstrapped whooping crane annual survival for each iteration of the repeated cross‐validated LASSO procedure so predicted population growth would incorporate uncertainty and variability in survival and predicted recruitment.

If management options that directly manipulate recruitment are unavailable, management options may exist to manipulate annual survival. Therefore, we estimated the annual survival ($\hat{\overset{¯}{S}}$) required to avoid population decline or avoid population growth from falling below average (i.e., *λ* ≥ 1.035) for each scenario where $\hat{\overset{¯}{S}}$ is $1/(1+\hat{\overset{¯}{R}})$ (Butler et al., 2014).

## 3 Results

The whooping crane population has grown at approximately 3.5% annually (geometric mean, *n *=* *61, *SD* = 10.7) since 1950 but varied from −19.4% to 33.3%. Using all survey data, recruitment of juvenile birds ($\hat{R}$; ratio of hatch‐year to after‐hatch‐year birds) into the winter population of whooping cranes ranged from 0 to 0.4 (geometric mean = 0.145, *n *=* *61, *SD* = 0.090). Annual whooping crane survival ($\hat{S}$) ranged from 0.645 to 1.0 (geometric mean = 0.906, *n *=* *60, *SD* = 0.060).

### 3.1 Addressing bias in recruitment estimates

We found that recruitment was biased high for years with few surveys. Specifically, it was inversely related to the number of surveys conducted such that an additional survey produced a 3.6% decline in recruitment ($\hat{\beta }$ = −0.036, *SE* = 0.010, *p *<* *.001). This occurred because detection of a whooping crane group was 55.8% (Strobel & Butler, 2014) resulting in the cumulative detection probably not exceeding 99% until six surveys were completed. Therefore, we removed years in which the number of surveys conducted was fewer than six (*n *=* *6). Once years with <6 surveys were removed, recruitment was no longer associated with the number of surveys conducted ($\hat{\beta }$ = −0.016, *SE* = 0.012, *p *=* *.119). Recruitment during years with ≥6 surveys ranged from 0 to 0.31 (geometric mean = 0.137, *n *=* *55, *SD* = 0.074).

### 3.2 Modeling recruitment and survival with LASSO

The repeated cross‐validated LASSO procedure suggested four variables were important to predicting recruitment (selected by >95% of iterations; Table 2). Sunspot number (*n *=* *55, mean = 94.8, *SD* = 69.1, min = 4.2, max = 225.1) was the most important predictor of recruitment and the model indicated that as sunspots increased, recruitment declined (SSN; Table 2). Precipitation during autumn migration in the northern U.S. Great Plains (ZNDX.NEDA.1011; *n *=* *55, mean = 0.49, *SD* = 1.26, min = −1.60, max = 3.74) was the second most important predictor (based on standardized $\hat{\beta }$; Table 2). As precipitation increased during autumn migration, recruitment declined. Conditions on the breeding grounds were also important. Recruitment increased as more days remained below freezing during winter (DX32.WBNP.1103; *n *=* *55, mean = 0.91, *SD* = 0.05, min = 0.74, max = 0.99), and it declined as precipitation increased during the breeding season (TPCP.WBNP.0409; *n *=* *55, mean = 23.4, *SD* = 5 = .8, min = 9.7, max = 41.6; Table 2). Two other variables were of lesser certainty in their importance (selected by ≈75% of iterations; Table 2). Greater precipitation on the wintering grounds prior to spring migration (ZNDX.ANWR.1103; *n *=* *55, mean = 0.04, *SD* = 1.19, min = −2.44, max = 3.57) was associated with increased recruitment (Table 2). Increased precipitation on the breeding grounds during winter (TPCP.WBNP.1103; *n *=* *55, mean = 8.7, *SD* = 2.5, min = 4.3, max = 15.1) was associated with decreased recruitment (Table 2). The repeated cross‐validated LASSO procedure suggested none of the variables were important to predicting annual survival.

**Table 2:** Variables predicting recruitment of juvenile whooping cranes into the winter population

Predictor variable	Proportion of models^a^	Standardized $\hat{\beta }$	$\hat{\beta }$	*SE*	*p*
SSN	1.000	–0.166	–0.002	0.001	<.001
DX32.WBNP.1103	0.999	0.053	1.068	0.313	.001
ZNDX.NEDA.1011	0.984	–0.072	–0.057	0.021	.005
TPCP.WBNP.0409	0.960	–0.042	–0.007	0.003	.022
ZNDX.ANWR.1103	0.769	0.013	0.011	0.009	.254
TPCP.WBNP.1103	0.740	–0.028	–0.011	0.011	.324
DX32.WBNP.0409	0.286	–0.008	–0.264	0.510	.604
TPCP.SASK.04	0.264	–0.005	–0.004	0.008	.623
DT32.SASK.0910	0.247	0.006	0.076	0.160	.632
TPCP.SASK.0910	0.184	0.001	0.000	0.001	.692
PDSI.NEDA.04	0.170	–0.004	–0.002	0.005	.731
ZNDX.NEDA.04	0.117	0.002	0.001	0.004	.771

a Modeled recruitment (*n *=* *55) using LASSO regression and tenfold cross‐validation to select the optimal model. This procedure was repeated 1,000 times and proportion of times a variable was selected was recorded. Acronyms are defined in Table 1.

### 3.3 Modeling weather variables

The proportion of days that remained below freezing during winter on the breeding grounds (DX32.WBNP.1103) decreased as the sunspot number and natural log of atmospheric CO_2_ concentration increased (Table 3). If sunspot number was at the modern grand maximum (269.3) and CO_2_ concentration was 500 ppm, this model predicts the proportion of days that remained below freezing (DX32.WBNP.1103) would likely be 0.765 (*SE* = 0.042) which is within the range of values observed for this variable historically. On the northern U.S. Great Plains during October–November, precipitation (ZNDX.NEDA.1011) increased log‐linearly with atmospheric CO_2_ concentration (Table 3). If CO_2_ concentration was 500 ppm, this model predicts precipitation during southern migration through the northern U.S. Great Plains (ZNDX.NEDA.1011) would likely be 2.71 (*SE* = 0.88) which is also within the range of values observed for this variable historically. Neither atmospheric CO_2_ concentration nor sunspot number was associated with total precipitation on the breeding grounds during April–September (TPCP.WBNP.0409; Table 3).

**Table 3:** Weather variables that were associated with recruitment of juvenile whooping cranes into the winter population were modeled as functions of atmospheric CO_2_ concentration and/or sunspot number

Response^a^		Intercept		Sunspot no.		log CO_2_	
	*n*	$\hat{\beta }$	*SE*	$\hat{\beta }$	*SE*	$\hat{\beta }$	*SE*
ZNDX.NEDA.1011	64	–34.768	14.026			6.031	2.396
DX32.WBNP.1103	65	14.151	3.209	–0.002	0.001	–1.986	0.545
TPCP.WBNP.0409	65	23.327	0.693				

a All weather variables were modeled with simple linear regression except DX32.WBNP.1103 which was modeled using logistic regression. Acronyms are defined in Table 1.

### 3.4 Relationships with nesting pond depth

Mean water depth in nesting ponds ranged from 14.3 to 27.8 cm (mean = 21.4 cm, *SD* = 4.21). Mean water depth in nesting ponds exhibited a relationship with sunspot number ($\hat{\beta }$ = −0.038, *SE* = 0.009, *t *= −4.172, *p *=* *.001), proportion of days that remained below freezing on the breeding grounds during November–March ($\hat{\beta }$ = 44.838, *SE* = 15.078, *t *=* *2.974, *p *=* *.012), and total precipitation on the breeding grounds during November–March ($\hat{\beta }$ = −0.555, *SE* = 0.265, *t *= −2.096, *p *=* *.058). Whooping crane recruitment increased as pond depth increased (odds ratio = 1.097, $\hat{\beta }$ = 0.093, *SE* = 0.021, *p *<* *.001).

### 3.5 Population growth, recruitment, survival, and environmental change

**Figure 2:**
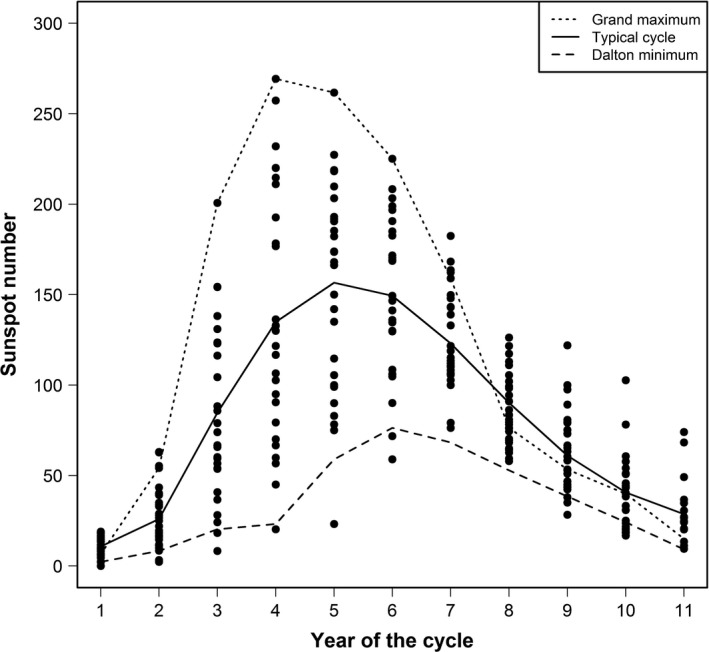
The solar cycle as indicated by sunspot number has varied over the last three centuries (1712–2007). The Dalton minimum occurred during 1811–1821 and modern grand maximum occurred during 1954–1964. We estimated the typical cycle as a sixth‐order polynomial of the number of years since a cycle began

**Figure 3:**
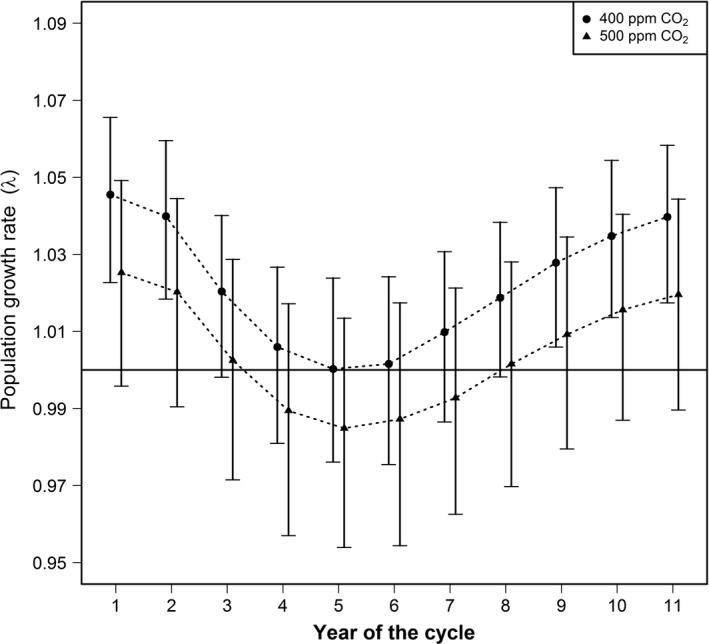
Predicted whooping crane population growth for two atmospheric CO_2_ concentration scenarios during a typical solar cycle (horizontal line indicates 0% population growth, λ = 1)

**Figure 4:**
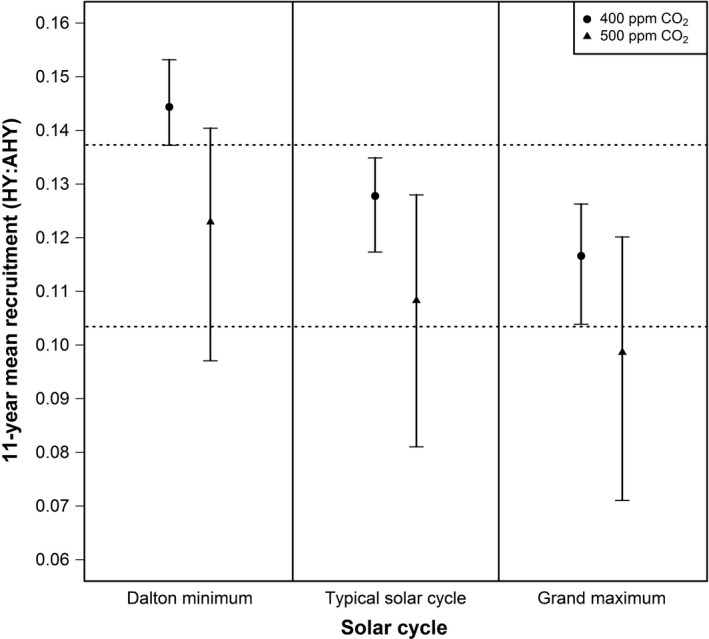
Predicted juvenile recruitment (ratio of hatch‐year [HY] to after‐hatch‐year [AHY] birds) of whooping cranes during an 11‐year solar cycle for two atmospheric CO_2_ concentration scenarios. Top dotted line represents long‐term mean recruitment (0.137) and bottom dotted line represents required recruitment (0.103) needed to maintain a stable population (λ ≥ 1) given the long‐term mean annual survival of 90.6%. Points represent mean predictions and vertical bars 95% confidence intervals

We used an 11‐year period for the typical sunspot cycle and estimated it as a sixth‐order polynomial of the number of years since a cycle began (model selected using AIC; min sunspot number = 10.8, max sunspot number = 156.6; Figure 2). We used this typical cycle, the Dalton minimum cycle (1811–1821; Figure 2), and the modern grand maximum cycle (1954–1964; Figure 2) to predict future whooping crane recruitment and population growth for two atmospheric CO_2_ concentration scenarios (i.e., 400 and 500 ppm). Whooping crane population growth fluctuates inversely to the solar cycle, resulting in greater population growth during years of reduced solar activity (Figure 3). At the current atmospheric CO_2_ concentration (≈400 ppm), years with negative growth are likely rare during a typical solar cycle (Figure 3). However, our model predicts years with negative growth become more common in the future (500 ppm CO_2_), as 4 years of the typical solar cycle have negative population growth (i.e., λ  <  1; Figure 3). Average recruitment is predicted to fall below the long‐term average (0.137) in all the scenarios, except one: a solar cycle similar to the Dalton minimum with 400 ppm CO_2_ (Figure 4). Indeed, at 500 ppm CO_2_ during a solar cycle similar to the modern grand maximum, mean recruitment lies below the amount required for population growth (Figure 4).

**Figure 5:**
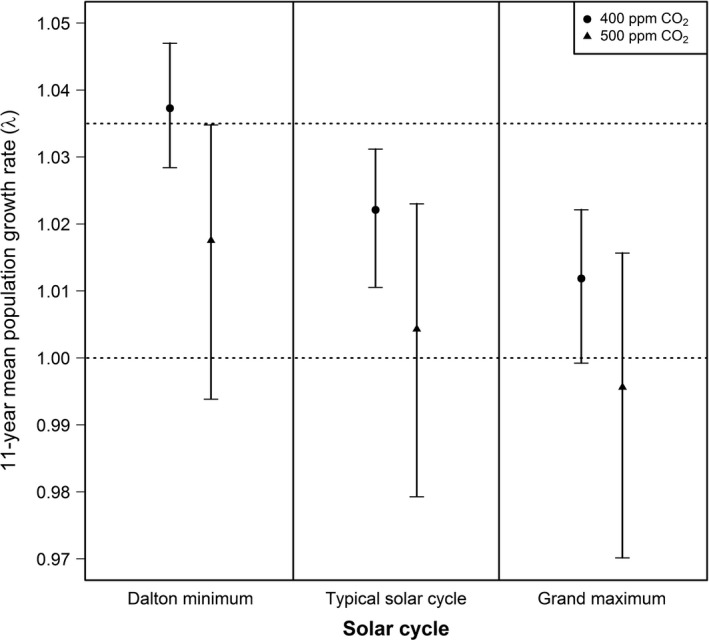
Predicted whooping crane population growth during an 11‐year solar cycle for two atmospheric CO_2_ concentration scenarios. Top dotted line represents long‐term mean population growth of 3.5%. Points represent mean predictions and vertical bars 95% confidence intervals. Only a solar cycle similar to the Dalton minimum with ≤400 ppm CO_2_ will likely maintain population growth at or above the long‐term mean of 3.5%

Regardless of the solar scenario, our model predicts reduced whooping crane population growth if atmospheric CO_2_ concentration continues to increase (Figure 5). Whooping crane population growth likely falls below the long‐term average (3.5%) for all solar conditions examined with 500 ppm CO_2_ (Figure 5). Further, the population will likely decline by −0.44% (95% CI = −2.98% to 1.57%) if a solar cycle similar to the modern grand maximum occurs in the future (Figure 5).

**Figure 6:**
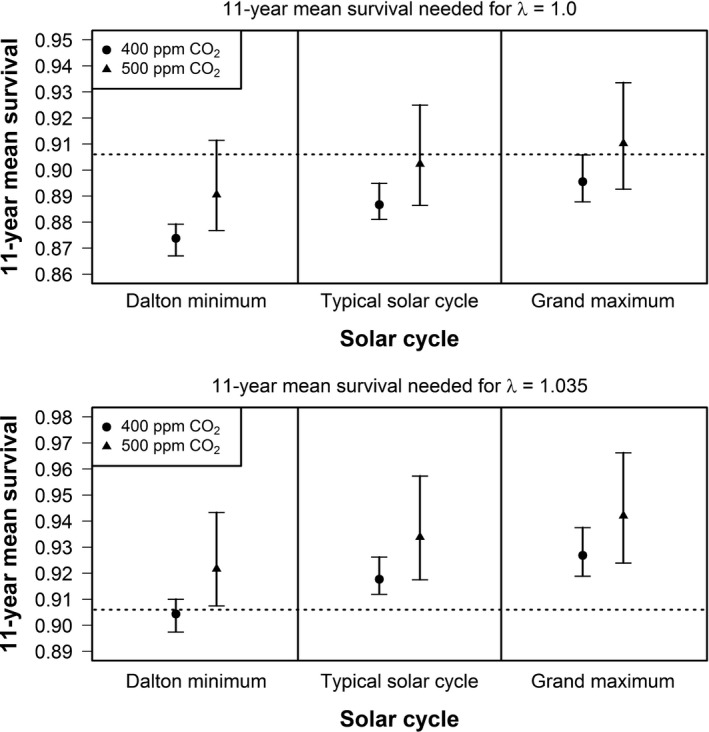
Predicted whooping crane survival needed to maintain 0% population growth and 3.5% population growth (long‐term mean) during an 11‐year solar cycle for two atmospheric CO_2_ concentration scenarios. Dotted line represents long‐term mean annual survival of 90.6%. Points represent mean predictions and vertical bars 95% confidence intervals

We found the past survival rate of 90.6% was likely sufficient to maintain a stable population (λ  ≥  1) for all but one scenario: a solar cycle similar to the modern grand maximum with 500 ppm CO_2_ (Figure 6). In this scenario, our model predicts annual survival would need to be increased to 91.0% (95% CI = 89.3%–93.4%; Figure 6). If the past population growth rate of 3.5% is to be maintained, then all scenarios with 500 ppm CO_2_ would require increasing annual survival above its long‐term mean (Figure 6). For example, during a typical solar cycle with 500 ppm CO_2_, our model predicts annual survival would need to be increased to 93.4% (95% CI = 91.8%–95.7%) to maintain 3.5% population growth (Figure 6).

## 4 Discussion

### 4.1 Environmental conditions and recruitment

Heightened solar activity increases the amount of radiation striking the Earth's surface (Krivova et al., 2010). This raises atmospheric temperatures and alters weather patterns thereby influencing plant growth, surface hydrology, and faunal responses (Klvana et al., 2004; Krebs et al., 2001; Marques, Short, et al., 2014; Stager, Ruzmaikin, Conway, Verburg, & Mason, 2007). Solar activity, as indexed by the sunspot number, had the greatest magnitude of effect on whooping crane recruitment (based on Standardized $\hat{\beta }$; Table 2), having ≥2.3 times more effect than any other variable. The mechanism(s) describing how sunspot activity affects whooping crane recruitment remains unknown. Unraveling similar correlations between sunspots and other cyclical biological phenomena within the boreal ecosystem, and identifying mechanism(s) that generate these observed relationships also remains elusive (Krebs et al., 2001; Sinclair & Gosline, 1997).

The amount of precipitation in the northern U.S. Great Plains during the autumn migration was the second most important predictor. This variable had ≥36% the influence of the remaining weather variables. Recruitment declined as the amount of precipitation increased during autumn migration. We hypothesize that juveniles are more vulnerable to mortality or abandonment during inclement weather. As juveniles have greater energy expenditure during migration than adults (Rotics et al., 2016) and are inexperienced foragers, inclement weather may reduce foraging opportunities (Clark, 2009), predisposing juveniles to greater morality risk. Further, inclement weather conditions can increase risk of collision for migrating birds with power lines or other infrastructure (Marques, Batalha, et al., 2014) and juvenile cranes are more vulnerable to collisions than adults (Brown & Drewien, 1995; Chavez‐Ramirez & Wehtje, 2012; Ward & Anderson, 1992).

On the breeding grounds, recruitment increased with more days remaining below freezing during winter. We also observed that nesting pond depth decreased as the number of days remaining below freezing during winter decreased. The potential mechanism behind the association between recruitment and the number of freezing days during winter may be that fewer cold days (<0°C) reduces the amount of time the active groundwater layer remains frozen. Frozen earth retains pond water into the nesting period, with thinner layers more easily breached by the water trapped above (Yoshikawa & Hinzman, 2003). Thawing of the ground allows water to permeate the soil, resulting in reduced pond depth, size, and longevity. Presumably, adult whooping cranes nest within ponds to reduce predation on themselves, eggs and hatchlings (Kuyt et al., 1992; Timoney et al., 1997). If ponds dry too quickly, nests may become more vulnerable to terrestrial predators, thereby lowering hatchling survival resulting in reduced recruitment.

Precipitation on the breeding grounds during winter, though much less influential than other weather variables, reduced recruitment. Increased precipitation, likely in the form of snow, acts as an insulator maintaining soil warmth, reducing the thickness of frozen earth and thereby reducing water depth in nesting ponds (Yoshikawa & Hinzman, 2003). Corroborating this, we observed nesting pond depth declined as winter precipitation increased.

Increased precipitation during the breeding season on the breeding grounds was associated with reduced recruitment. We hypothesize that increased precipitation during incubation causes nest flooding and failure (Ivey & Dugger, 2008; Layne, 1983). Furthermore, increased rainfall is more apt to soak nesters and hatchlings, thereby increasing their risk of illness or death (Chavez‐Ramirez & Wehtje, 2012; Layne, 1983).

Variables associated with recruitment primarily operate on the breeding grounds or during southern migration across the northern U.S. Great Plains. The variables operating in other locations were much less influential. For example, precipitation on the wintering grounds prior to spring migration and breeding was associated with increased recruitment. This suggests that whooping cranes do not exhibit a complete income breeding strategy (Stephens et al., 2009). Instead, breeding propensity or other reproductive parameters appear partially controlled by conditions prior to migration. However, precipitation on the wintering grounds was at least 69% less influential than the other weather variables already discussed (Table 2).

### 4.2 Potential impacts of climate change on recruitment and population growth

Elevated concentrations of atmospheric CO_2_ warm the Earth, reducing the proportion of days below freezing on the breeding grounds (i.e., increased temperature; Lemmen, Warren, Lacroix, & Bush, 2008) and increasing precipitation on the northern U.S. Great Plains during autumn (Melillo, Richmond, & Yohe, 2014). Given these relationships, we substituted the amount of atmospheric CO_2_ for these variables to evaluate the potential effects of CO_2_ concentration on recruitment.

Our model predicts recruitment declines as the magnitude of the solar cycle moves from the Dalton minimum to the modern grand maximum, or as atmospheric CO_2_ concentrations increase from 400 to 500 ppm (Figure 4). The influence of sunspots and atmospheric CO_2_ on recruitment is comparable, although sunspots have a greater effect. For example, at 400 ppm CO_2_ concentration, the difference in average population growth between the Dalton minimum (3.7% mean growth; Figure 5) and the modern grand maximum (1.2% mean growth; Figure 5) is 2.5% (95% CI mean difference = 1.3%–3.8%). Hence, a solar cycle similar to the grand maximum would result in ≈68% less population growth than a solar cycle similar to the Dalton minimum. Alternatively, during a typical solar cycle, the difference in average population growth between 400 ppm (2.2% mean growth; Figure 5) and 500 ppm CO_2_ concentration (0.4% mean growth; Figure 5) is 1.8% (95% CI mean difference = 0.3%–3.6%). Hence, as atmospheric CO_2_ concentration increases to 500 ppm, population growth will likely be reduced by ≈80% compared to the current CO_2_ concentration.

Only one scenario, a solar cycle similar to the Dalton minimum with 400 ppm CO_2_ concentration, results in population growth commensurate with the long‐term average (i.e., >3.5% growth; Figure 5). This scenario results in no simulations indicating a population decline. All other scenarios portend a future with whooping crane population growth likely falling below the long‐term average (Figure 5). Population growth will likely be reduced by half during a solar cycle similar to the Dalton minimum with 500 ppm CO_2_, suggesting downlisting and recovery may take two times longer. The most likely future scenario, a typical solar cycle with 500 ppm CO_2_, suggests population growth will likely be minimal (0.4% mean growth which is an eighth of the long‐term average), with 31% of the simulations indicating a population decline. These results suggest downlisting and recovery may require eight times longer than conditions without climate change (Butler et al., 2013). If the scenario of a solar cycle similar to the grand maximum with 500 ppm CO_2_ concentration occurs, then 63% of the simulations indicate population declines, likely precluding downlisting and recovery in the foreseeable future.

The sunspot numbers that compose the scenarios described above were observed within the data used (min = 4.2, max = 225.1) to build our models, with the exception of the minimum sunspot number observed during the Dalton minimum (2.3) and the two maximum sunspot numbers observed during the modern grand maximum (261.7 and 269.3). Although the 500 ppm CO_2_ concentration is beyond the range of the data upon which our models were built, the relationships we developed between weather variables (DX32.WBNP.1103, ZNDX.NEDA.1011) and CO_2_ concentration assumed a log‐linear relationship which results in conservative estimates of the potential magnitude of change. Further, the predictions at 500 ppm CO_2_ concentration and the modern grand maximum for these weather variables were within the range of values observed for the variables historically (see results). Hence, our predictions about potential impacts of climate change on recruitment and population growth are tempered and reasonable.

### 4.3 Management intervention to improve recruitment

Recruitment should remain at or above 0.103 to maintain stable to increasing population growth (λ ≥ 1; Figure 4). If the scenario of a solar cycle similar to the grand maximum with 500 ppm CO_2_ occurs, recruitment may decline to 0.099 (95% CI = 0.071–0.120; Figure 4) and management intervention on the breeding grounds would likely have to result, on average, in an additional 0.5 juveniles per 100 adults to avoid population decline. To maintain long‐term average population growth (3.5%), recruitment should remain at or above 0.137 (Figure 4). The only scenario predicted to achieve this is 400 ppm CO_2_ concentration during a solar cycle similar to the Dalton minimum (Figures 4 and 5). Other scenarios, such as a typical solar cycle or modern grand maximum with 500 ppm CO_2_ concentration suggests average recruitment may decline to 0.108 (95% CI = 0.081–0.128) and 0.099 (95% CI = 0.071–0.120; Figure 4), respectively. Thus, any management intervention on the breeding grounds would likely have to result, on average, in an additional 3 to 4 juveniles recruited per 100 adults to maintain the long‐term population growth.

These targets appear achievable. Potential management strategies include predator management (Littlefield, 2003), the addition of nesting platforms or structures (Maggiulli & Dugger, 2011; Miller, Grand, Fondell, & Anthony, 2007), and manipulation of nesting pond depths. While it has been suggested that management intervention on Wood Buffalo National Park is infeasible given its remoteness and lack of infrastructure (Wilson et al., 2016), these challenges would need to be overcome to recover whooping cranes. Without management intervention, the required amount of time to achieve population goals for downlisting and recovery likely becomes greatly extended or potentially impossible, given our model's predictions.

How climate change may manifest in the predator–prey cycle remains unclear. Lynx declines have been linked to climate warming (Yan et al., 2013), yet we do not know the ecological ramifications of this outcome, or climate effects on other predators. Other complex interactions associated with ponds drying and increased vulnerability to nest and fledgling predation could also decrease recruitment even if predator densities remained similar or declined. Furthermore, increased temperatures may result in predator populations becoming less cyclic and more generalized in prey selection (Scott & Craine, 1993).

### 4.4 Management intervention to improve annual survival

If management intervention cannot improve recruitment, maintaining long‐term average population growth (3.5%) likely requires increases in annual adult survival in all but one of the scenarios (solar cycle similar to the Dalton minimum with 400 ppm CO_2_; Figure 6). For instance, the scenario of a typical solar cycle with 500 ppm CO_2_, annual adult survival should be increased from 90.6% (long‐term geometric mean; *SD* = 0.060) to 93.4% (95% CI = 91.8%–95.7%; Figure 6) to maintain the long‐term average population growth. At the current population of ≈340 whooping cranes, this is equivalent to, on average, 9 to 10 fewer adult mortalities per year. However, none of the variables we examined were important to predicting survival and many management actions are already underway to reduce adult mortality (e.g., signage to inform hunters, marking of power lines in strategic locations). Past mortality from power line collisions and incidental or illegal harvest is rare (<1 bird/year succumbs to these mortality causes; Stehn & Haralson‐Strobel, 2014). Thus, little gains in annual survival can currently be expected from these management actions.

Long‐term annual adult survival is 90.6% and likely remains sufficient to maintain population stability (0% growth) across all solar cycle and CO_2_ concentration scenarios except during a solar cycle similar to the grand maximum with 500 ppm CO_2_ (Figure 6). Given this scenario, annual survival needs to reach 91.0% (95% CI = 89.3%–93.4%; Figure 6) to avoid a population decline. In a scenario where a typical solar cycle occurs with 500 ppm CO_2_, annual adult survival could decline to 90.2% (95% CI = 88.7%–92.5%; Figure 6) and still avoid population decline. Hence, given a typical solar cycle with 500 ppm CO_2_ and the current population of ≈340 whooping cranes, on average, 1 to 2 additional mortalities per year could result in a declining population.

### 4.5 Synthesis

This whooping crane population will likely continue experiencing periodic and predictable years of low recruitment and growth, as sunspots form the primary driver of their approximate decadal population cycle. Climate change likely threatens this population by 2050 when CO_2_ concentrations are expected to reach 500 ppm, as our model predicts population growth can become negative during any solar cycle (unlike 400 ppm CO_2_; Figure 5). Hence, science has roughly three decades to reveal the on‐the‐ground mechanisms relating solar activity and atmospheric CO_2_ with recruitment (i.e., proximate causes) and to design approaches for mitigating the adverse effects. Alternatively, other management actions could increase annual survival to avoid population declines. Optimistically, increasing recruitment and survival so the population continues growing requires only 1–4 additional recruits or survivors per year. Survivorship is the tougher to address, as many of the actions are continental in scope and multifaceted (e.g., power line marking, wind turbine curtailment, hunter education and law enforcement, etc.).

Recruitment affects whooping crane population growth most (Butler et al., 2014; Wilson et al., 2016), and our model predictions show that climate change may imperil it. Hence, any perceptions that whooping cranes remain secure in Canada, and can remain free of active management, are false. If conserving whooping cranes in North America remains an important goal, then attention must center on understanding mechanisms limiting recruitment. There appears to be a connection between recruitment, breeding pond hydrology, and predation. Already, the numbers of boreal ponds and their temporal longevity during summer are declining (Carroll et al., 2011; Smith et al., 2005). Unless trends of increasing atmospheric CO_2_ are abated, science must determine how to mitigate the likely reduction in recruitment by 2050. Future monitoring and research should focus on relationships of depth and seasonal longevity of nesting ponds with nest success and causes of nest failure. Additionally, fledgling mortality and its causes on the breeding grounds and during southern migration should be investigated. The results of this work could steer conservation actions toward long‐term sustainability and protection of whooping cranes in North America. If such efforts prove unsuccessful, then the only wild population of this species must adapt or likely face extinction.

## Conflict of Interest

None declared.
